# Psychiatric comorbidity: fact or artifact?

**DOI:** 10.1007/s11017-015-9321-0

**Published:** 2015-02-01

**Authors:** Hanna M. van Loo, Jan-Willem Romeijn

**Affiliations:** 1 Interdisciplinary Center Psychopathology and Emotion Regulation (ICPE), Department of Psychiatry, University Medical Center Groningen, Hanzeplein 1, PO Box 30.001, 9700 RB Groningen, The Netherlands; 2 Faculty of Philosophy, University of Groningen, Oude Boteringestraat 52, 9712 GL Groningen, The Netherlands; 3 Philosophy Department, University of Johannesburg, Johannesburg, South Africa

**Keywords:** Comorbidity, Psychiatric disorders, DSM, Disease classification, Conventionalism

## Abstract

The frequent occurrence of comorbidity has brought about an extensive theoretical debate in psychiatry. Why are the rates of psychiatric comorbidity so high and what are their implications for the ontological and epistemological status of comorbid psychiatric diseases? Current explanations focus either on classification choices or on causal ties between disorders. Based on empirical and philosophical arguments, we propose a conventionalist interpretation of psychiatric comorbidity instead. We argue that a conventionalist approach fits well with research and clinical practice and resolves two problems for psychiatric diseases: experimenter’s regress and arbitrariness.

## Introduction

This article investigates the nature of comorbidity among psychiatric diseases, and considers how this reflects on psychiatric disease classification. Psychiatric disorders as described in the Diagnostic and Statistical Manual of Mental Disorders (DSM) are a topic of continuing debate.^1^ This debate reached a climax with the development of the fifth edition (DSM-5) [1], which provoked considerable controversy in the field of psychiatry, as well as in the broader community (e.g., [2–4]). The controversy touched on a wide range of issues: the transformation of normal emotional experiences to disorders, the pros and cons of defining disorders in dimensions instead of categories, the influence of the pharmaceutical industry on the development of new categories, and so on. Many of these issues are inextricably connected to a fundamental question about the status of current psychiatric disorders, to wit, how should we interpret categories in the DSM? What kind of structures are they? Do they refer to something real, or are they rather the product of our own categorizing efforts?

We approach these questions by an analysis of the phenomenon of comorbidity in psychiatry, i.e., the presence of two or more mental disorders in one individual. Comorbidity is an important concern for professionals and researchers. It occurs frequently in psychiatry: as many as 45 % of patients satisfy the criteria for more than one disorder in the course of a year. Disorders that co-occur often are mood and anxiety disorders, such as major depressive disorder (MDD) and generalized anxiety disorder (GAD) [5]. In addition, comorbidity is associated with a more severe course of illness. Patients suffering from both MDD and GAD tend to have a poorer prognosis and a disproportionally higher functional disability when compared to patients suffering from only one disorder [6].

Comorbidity’s high prevalence and its influence on disease severity make it an important subject of study, certainly insofar as the aim of research is to improve the lot of psychiatric patients. In addition, comorbidity patterns have led to significant theoretical debates on the nature of disease classification in psychiatry. The debate we focus on in this article concerns the artificiality or reality of high rates of comorbidity. Some argue that comorbidity is an artifact of our current diagnostic system, caused by all types of classification choices [7–9]. Other researchers in psychiatry contend that psychiatric comorbidity is indicative of something genuine about the nature of psychiatric disease by pointing to commonalities in the causal background of different disorders (cf. [10], and to some extent [11, 12]). The discussion about comorbidity is thus reminiscent of the main positions on the epistemological and ontological status of psychiatric disorders in general, which can be divided into a constructivist and realist camp.

The aim of this article is to scrutinize the phenomenon of comorbidity in psychiatry, and thereby, shed light on the nature of psychiatric disease classification. Do those categories reveal something real and robust about the psychiatric domain? Or are they rather the result of our own way of organizing the subject matter? Rather than opting for either of these extreme positions, we argue for a conventionalist position, which escapes the opposition above: categories in the DSM offer a robust picture of the world of psychiatric disorders, yet, they do so relative to a number of conventions. This way of viewing psychiatric disorders resolves two particular problems for the DSM regarding definitional circularity and arbitrariness. Moreover, we argue that conventionalism might benefit psychiatric science by clarifying the definitional status of the DSM without discarding current empirical findings as artificial.

The article is set up as follows. We start by reviewing the debate over psychiatric comorbidity, showing that this debate can be structured by grouping authors according to constructivist and realist sympathies. We then illustrate, by analyzing comorbidity data from the Netherlands Mental Health Survey and Incidence Study (NEMESIS) [13], that both types of explanation are insufficient to account for the high rates of comorbidity in psychiatry. Using our empirical example, we present and elaborate conventionalism regarding mental illness as an alternative. We illustrate this position by referring back to a debate in the philosophy of science over the ontological and epistemological status of geometrical descriptions of physical space [14–16]. Furthermore, we discuss the definitional circularity and related problems for the DSM. The upshot of the article is an improved understanding of comorbidity in psychiatry and of psychiatric disease classification in general.

## The DSM and comorbidity

The DSM is the most important classification system in psychiatry: it provides definitions for psychiatric disorders and is extensively used in clinical practice and research.^2^ The new, fifth edition describes a few hundred psychiatric disorders varying from schizophrenia, depressive disorders, and dementia to feeding and eating disorders [1]. Most psychiatric disorders are defined in terms of a set of symptoms, of which a certain number are necessary and sufficient.

### Comorbidity as dependent on classification choices

Specific definitional choices made in the DSM play a central role in the theoretical debate on comorbidity, as a broad range of these choices are thought to influence the rates of comorbidity. This has led some authors to claim that comorbidity rates are overstated and, perhaps, entirely artificial [7, 8]. An often mentioned example of those choices is the continuously increasing number of diseases in the DSM. The idea is that the proliferation of psychiatric categories increases comorbidity rates [7]. Second, comorbidity rates are thought to increase by lowering the necessary number of criteria to be satisfied for diagnoses, the so-called “threshold” [8]. In the case of anorexia nervosa, the number of criteria for the diagnosis in the DSM-5 is reduced from four to three necessary symptoms, which invokes the idea that more individuals will suffer from anorexia nervosa, and thus increase comorbidity rates [1]. Third, the progressive reduction of exclusionary rules is assumed to increase rates of comorbidity [7, 9]. Some disease definitions contain exclusionary rules that exclude the diagnosis in case of the presence of certain criteria. For example, the diagnosis MDD is excluded if the symptoms for a mixed episode are met, or if the symptoms derive from substance abuse or another medical condition [1].

A fourth, and often discussed, phenomenon is the presence of symptom overlap: some symptoms are part of the defining sets of more than one disorder, and are thus overlapping. For instance, the symptoms of sleep disturbance, difficulty in concentrating, and fatigue are part of the defining sets of both MDD and GAD in the DSM-5. Overlapping symptoms are thought to increase the co-occurrence of diseases with similar symptoms in their defining sets [18, 19]. A last point of concern is the non-specificity of defining symptoms. Symptoms are non-specific for a disease if they also occur frequently in individuals without this particular disease. For example, all patients suffering from depression are thought to suffer from feeling gloomy, sleeping badly, etc., but these symptoms also occur regularly in persons with other emotional disorders in which these symptoms are not included as defining criteria [18]. The addition of non-specific (“accessory”) symptoms to define disorders is supposed to increase rates of comorbidity as well [8].

### Comorbidity as dependent on causality

Instead of ascribing comorbidity to classification choices, other authors have emphasized the real character of psychiatric comorbidity, by referring to common causal structures (e.g., [10–12, 19] ). They stress that if there is a common causal structure for two diseases, those two diseases will co-occur more often than expected by chance. Consequently, comorbidity is seen as a signal that current diagnoses do not track all the underlying causes, and hence as a guide for improving our classificatory system. In other words, the high rates of comorbidity in psychiatry are believed to indicate the causal connections between disorders as they are currently defined.

**Fig. 1:**
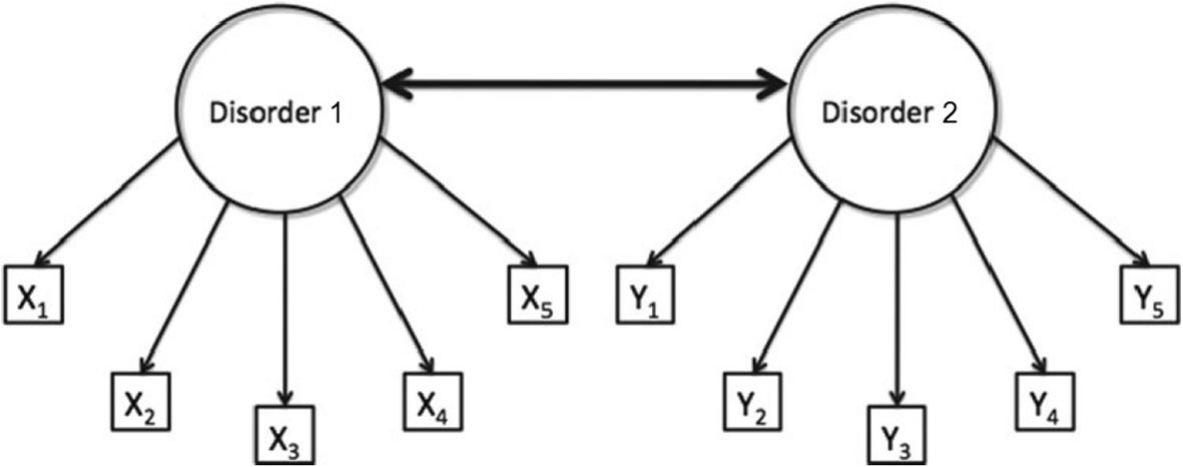
A model of comorbidity between disorders 1 and 2, under the standard assumptions of latent variable modeling. The *circles* represent the disorders (i.e., latent variables) and the *rectangles* represent the observable core symptoms of those disorders (i.e., X1–X5 for disorder 1, and Y1–Y5 for disorder 2). In this model, comorbidity is viewed as a correlation between the latent variables, visualized by the thick bidirectional edge between disorders 1 and 2 (Figure from Cramer et al. [21] with minor adjustments; reprinted with permission)

Recently, a debate has developed over the level at which these causal links between psychiatric disorders occur. In psychometrics, disorders are standardly approached as latent variable models. According to such models, correlations among the symptoms can be traced back to underlying constructs, i.e., variables that are latent and hence not directly observable. The comorbidity of two disorders can then be explained by a causal connection between the underlying constructs (Fig. 1).^3^ For example, the latent disorder GAD might cause the latent disorder MDD, or vice versa, and such relations between disorders can be measured by the development of depressed mood, anhedonia, and so on. In this setup, the symptoms themselves are supposed to be causally unconnected.

**Fig. 2:**
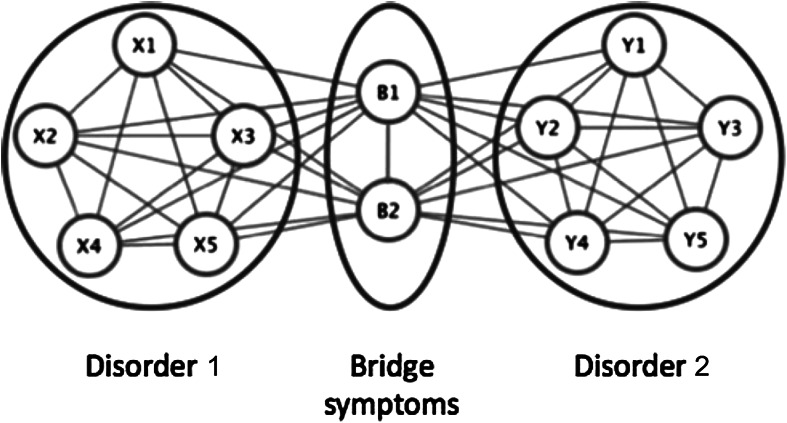
Comorbidity under a network approach. Disorder 1 consists of bidirectionally related symptoms X1–X5, and disorder 2 consists of symptoms Y1–Y5. Symptoms B1 and B2 are bridge symptoms that overlap between disorders 1 and 2. In this model, comorbidity arises as a result of direct relations between the bridge symptoms of two disorders (Figure from Cramer et al. [21] with minor adjustments; reprinted with permission)

Alternatively, psychiatric disorders can be modelled as networks in which symptoms are directly causally connected (Fig. 2) [21, 22]. In this understanding of psychiatric disorders, the level of latent variables is missing, and all the causal relations among disorders are realized in terms of those causal relations between symptoms. The presence of one symptom (say, insomnia) might stimulate the development of a host of connected symptoms (e.g., fatigue, concentration difficulties, and depressed mood). Because symptoms belonging to different diseases maintain causal ties, one disease will trigger the manifestation of another and hence increase comorbidity rates.

Summing up, according to both the traditional psychometric and the network models, comorbidity can be traced back to causal links between the disorders, although these links are localized on different levels. This supports the view that comorbidity reflects a real phenomenon in psychiatry.

### Arbitrariness and circularity

In short, one can interpret comorbidity in two different ways: either the comorbidity rates are determined by classification choices in the DSM and therefore are artificially high, or they result from causal relations between psychiatric disorders.^4^ One could ask: should psychiatrists interpret comorbidity as real or, rather, as the result of their constructions? To what extent are these two views, which one might somewhat tentatively call realist and constructivist, right in explaining comorbidity? Prima facie, the latter might seem the more attractive option. It seems undeniable that at least some comorbidities are the result of classification choices. Further arguments in favor of this constructivist reading of comorbidity derive from two closely connected problems for the DSM, which have to do with the idea that current diagnoses are arbitrary and do not cut nature at its joints, and with the definitional circularity that besets theory and measurement device. We briefly discuss these problems here.

First, consider the possible arbitrariness of the symptom sets as definitions for psychiatric disorders. Oftentimes, psychiatric disorders cannot be associated with distinct sets of symptoms. If we were to depict the empirical distribution of patients in an abstract space of symptom combinations, groups that suffer from MDD and GAD form a continuous whole. In other words, when it comes to the empirical facts about patient groups and the symptoms that they present, there is no clear “zone of rarity” that separates them. The question is whether it is sensible at all to speak of two separate disorders instead of one depression-anxiety disorder (cf. [24]). Absent zones of rarity are specifically problematic for advocates of causal disease models, as fuzzy disease boundaries seem to be at odds with the idea that diseases are identifiable bearers of causal relations among disorders [23, 25].

A related point concerns the apparent twofold function of the DSM. First, the structure of the DSM can be interpreted as a representation of the structure of psychiatric disorders, and hence, as a theory about what psychiatric disorders are [22]. But the same structure is also used as a measurement device intended to provide epistemic access to psychiatric disorders. So, definition and measurement of psychiatric disorders coincide exactly. The result of this double function is a circularity in the definition of the theoretical terms used in the DSM because the DSM indicates simultaneously what it is that one is measuring, and how one should go about measuring it. Now, in most empirical sciences, theory and device show a certain independence from one another, so that this circle can be broken. This is not unfortunately the case for the DSM, and this leads to a definitional circularity.^5^

### The status of the DSM

All in all, a causal reading of comorbidity might look somewhat unattractive. But despite these conceptual problems, many psychiatrists have the firm conviction that disorders are not arbitrary and that they can play a causal role. As we indicated, the two positions regarding comorbidity are related to what may be called the ontological status of psychiatric disease categories. The basic opposition is the one between constructivists and realists, and in this opposition, psychiatrists often lean towards the realist side.^6^ Despite the problems that beset realism about psychiatric disorders, one should not give up realist aspirations too soon.

In what follows, we first clarify the notion of comorbidity further, and argue that both positions—i.e., comorbidity as fact or artifact—are insufficient in their explanation of the phenomenon of comorbidity. Instead, comorbidity is the result of the interplay between both classification choices and population characteristics. We illustrate this in the following section by showing various simple disease models including their potential capacity for comorbidity, after which we analyze the actual comorbidity by using data from the Netherlands Mental Health Survey and Incidence Study. In the final section, we then put this view in a broader philosophical perspective, and apply it to psychiatric disease classifications more broadly.

## Comorbidity is the result of classification choices and population characteristics

To get a grip on psychiatric comorbidity, two elements are important: (1) how diseases are defined in terms of symptoms and (2) how frequently combinations of symptoms occur in a population.^7^ In this section, we introduce a diagrammatic representation in which both are visualized. The diagrams reveal that comorbidity is the result of the interplay between specifics of a population and the way diseases are modelled. This establishes an empirical argument against univocal explanations of comorbidity: comorbidity cannot be explained solely by reference to classification choices, and neither can it be fully explained by viewing the diseases as entities and by pointing to relations between them. The empirical study, rather, provides an argument for the adoption of a conventionalist view.

### Diagrammatic representation

**Fig. 3:**
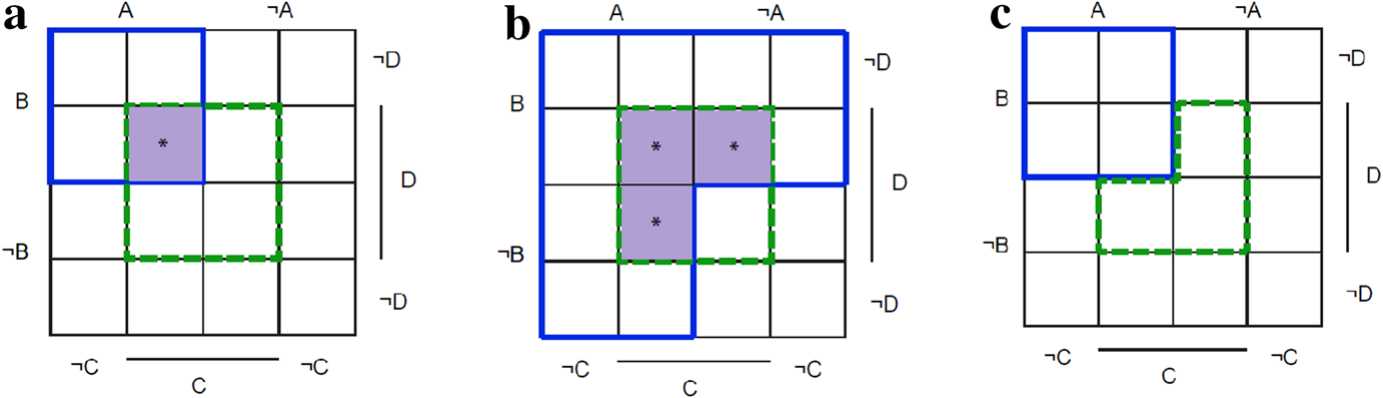
Different disease models and their potential for comorbidity. D_1_ *solid line*; D_2_ *dashed line*; *potential comorbid symptom combinations. **a** Two monothetic disease models (*D*_*1*_: *A* ∩ *B*; *D*_*2*_: *C* ∩ *D*). **b** *D*_*1*_ as a polythetic model (*D*_*1*_: *A* ∪ *B*; *D*_*2*_: *C* ∩ *D*). **c** *D*_*2*_ includes exclusionary rules (*D*_*1*_: *A* ∩ *B*; *D*_*2*_: ¬*A* ∩ ¬*B* ∩ *C* ∩ *D*)

In the diagrams of Fig. 3, symptoms are used as defining criteria for psychiatric diseases so as to mimic disease definitions in the DSM. Each symptom can be either absent or present. Every symptom combination consists of the total number of discerned symptoms, with every symptom indicated as absent or present. If *n* symptoms are defined, the total number of possible symptom combinations is 2^*n*^. One extreme of all combinations is 0 of *n* symptoms present; the other extreme is all symptoms present. The rest of the 2^*n*^ symptom combinations consist of all combinations of one or more and less than *n* symptoms present. In our example, the number of discerned symptoms is limited to four in total, denoted by *A*, *B*, *C*, and *D*.^8^

Diseases are defined in terms of the discerned symptoms. Different disease models are constructed in the diagrams of Fig. 3, illustrating some characteristics of disorders in the DSM. Figure 3a shows two monothetic diseases (*D*_*1*_ and *D*_*2*_), each consisting of two criteria (*D*_*1*_: *A* ∩ *B*; *D*_*2*_: *C* ∩ *D*). These diseases are monothetic since every symptom is necessary, and the set is sufficient for the diagnosis to be present. Four symptom combinations satisfy *D*_*1*_ (*A*, *B*, ¬*C*, ¬*D*; *A*, *B*,* ¬C*, *D*; *A*, *B*, *C*, ¬*D*; *A*, *B*, *C*, *D*) and four symptom combinations satisfy *D*_*2*_ (¬*A*, ¬*B*, *C*, *D*; ¬*A*, *B*, *C*, *D*; *A*, ¬*B*, *C*, *D*; *A*, *B*, *C*, *D*). In case of the presence of *A*, *B*, *C*, and *D*, there is comorbidity of *D*_*1*_ and *D*_*2*_ (*).

Figure 3b, c shows different variants of the basic model with several features occurring in the DSM: a polythetic model (Fig. 3b) and a model with an exclusionary rule (Fig. 3c). A polythetic disease consists of a set of criteria, of which patients have to satisfy at least a certain number, but no criterion of this set is necessary and sufficient. Exclusionary rules exclude the diagnosis in case of the presence of certain criteria. The number of symptom combinations satisfying both diseases is obviously dependent on definition choices. An increasing number of these potential comorbid symptom combinations substantiates the claim that adjustments of disease definitions result in higher comorbidity rates, and thus supports the idea that comorbidity is artificial. However, this is not necessarily true. To get a complete picture of how the rates of comorbidity depend on classification choices, the distribution of symptoms in the population must be taken into account. After all, if no individuals have the additional comorbid symptom combinations, rates of comorbidity will not change at all.

### NEMESIS study

To illustrate the influence of population characteristics on rates of comorbidity, we use data from the Netherlands Mental Health Survey and Incidence Study (NEMESIS). In NEMESIS, a representative sample was drawn from the general Dutch population between the ages 18 and 64 (*n* = 7,147). This sample was interviewed with the Dutch version of the Composite International Diagnostic Interview (CIDI). The CIDI is a structured psychiatric interview covering a very broad range of psychiatric complaints. Ultimately, this led to a dataset of 7,076 individuals. Bijl et al. [13] have provided a detailed description of the objectives and design of NEMESIS. From this data set, we selected eight symptoms for two analyses.

#### Analysis 1

For the first analysis, we studied the presence of symptoms of anxiety (*ANX*, i.e., feeling anxious, nervous, or worried), depressed mood (*DEP*, i.e., feeling depressed, gloomy, or in the dumps), insomnia (*INS*), and concentration difficulties (*CONC*) for the majority of a period of at least 2 weeks (or at least 4 weeks in case of anxiety) during the subject’s lifetime. These symptoms are part of MDD and GAD, which are diseases co-occurring very frequently [29]. With those symptoms, we aimed to find an example in which all symptom combinations occur regularly, and as a result, adjustments of disease models indeed change comorbidity rates. In the NEMESIS study, we determined the frequencies of each unique symptom combination in 7,072 individuals (*n* = 7,072, missing data in case of 4 individuals). All 16 possible combinations occurred regularly (min. 94, max. 2,390). 2,390 (33.8 %) individuals did not suffer from any symptoms during their lives, which was the most frequent finding. Notably, in case of symptoms being present, the most frequent symptom combination identified was all symptoms present (*n* = 1,084, 15.3 %). Least frequent was the combination of sleep problems and concentration problems, without depressed mood and without anxiety (1.3 %).

**Fig. 4:**
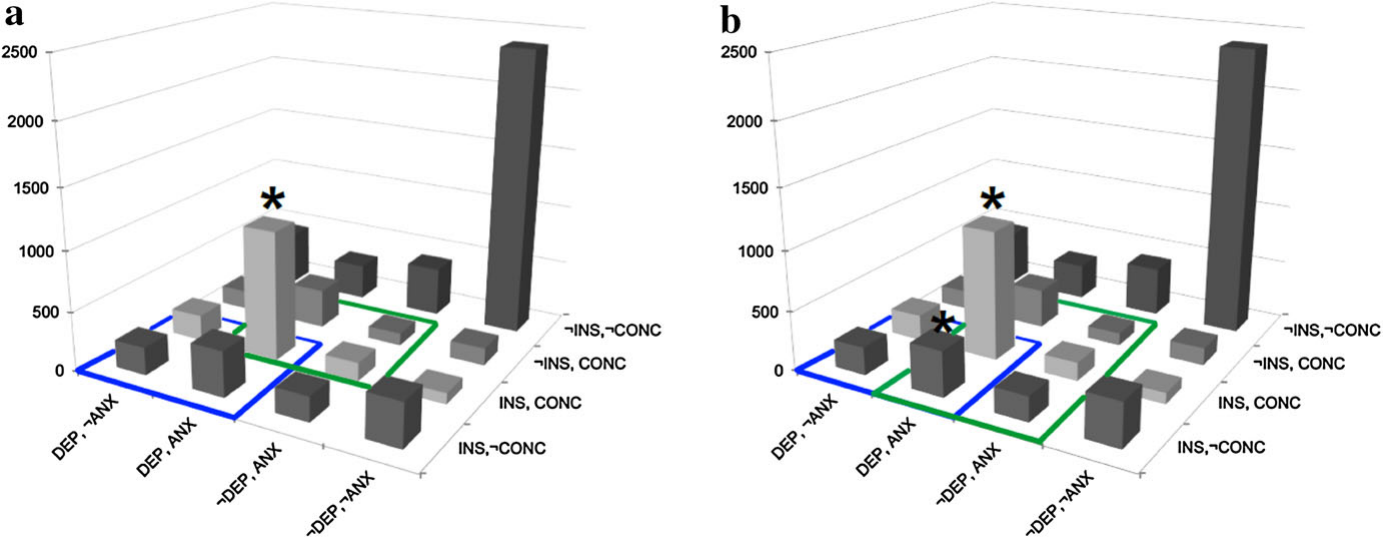
Histogram with responses on “Have you ever suffered from…?” Answers: *ANX*: anxiety, worrisome period of at least 1 month; *DEP*: depressed mood for at least 2 weeks; *INS*: insomnia for at least 2 weeks; *CONC*: concentration problems for at least 2 weeks (* marks comorbidity with rates of 43.1 % in **a** to 53.9 % in **b**)

Two simple monothetic disorders (*D*_*1*_ and *D*_*2*_) are constructed in Fig. 4a. *D*_*1*_ is defined as the combination of depressed mood and insomnia (*D*_*1*_: *DEP* ∩ *INS*); *D*_*2*_ consists of the monothetic set anxiety and concentration difficulties (*D*_*2*_: *ANX* ∩ *CONC*). In total, 1,923 patients satisfied *D*_*1*_; 1,675 patients satisfied *D*_*2*_. Of those patients, 1,084 patients satisfied *D*_*1*_ and *D*_*2*_ and, thus, suffered from comorbidity. In Fig. 4b, *D*_*2*_ is adjusted in a polythetic disorder (*D*_*2*_′): anxiety is still a required symptom, but in addition, a patient may suffer from concentration difficulties or sleep problems or both (*D*_*2*_′: *ANX* ∩ (*CONC* ∪ *INS*)). Therefore, two extra combinations of symptoms also satisfied this diagnosis (*ANX*, *INS* and *ANX*, *INS*, *DEP*), of which the latter implies comorbidity of *D*_*1*_ and *D*_*2*_. As a consequence, more individuals satisfied *D*_*2*_, and more individuals suffered from both disorders *D*_*1*_ and *D*_*2*_. Among the individuals satisfying a disorder, the percentage of comorbid patients increased from 43 to 54 %.

#### Analysis 2

For the second analysis, we selected symptoms of which we expected that certain symptom combinations were very unlikely to occur frequently. These symptoms were lifetime obsessions (*OBS*, i.e., persistent thoughts or urges that are experienced as intrusive and unwanted), compulsions (*COMP*, i.e., repetitive and unwanted behaviors such as checking locks of doors or washing hands), manic mood (*MAN,* i.e., a period of 2 days of feeling extremely cheerful leading to problems, worries among relatives, or diagnosis of mania), and drug use (*DR*, i.e., use of a specific drug more than five times).

Based on clinical experience, we expected especially combinations between obsessions or compulsions and drug use to be very rare. An analysis of the frequencies of all symptom combinations indeed led to very different results compared to Analysis 1. Of the 7,076 individuals (no missing data), a great majority of individuals did not report any of the four symptoms during their lifetime (83.2 %). The remaining 1,187 individuals reported at least one symptom. The most frequent symptom combination was drugs use as an isolated symptom (9.7 %).^9^ Furthermore, of the 16 possible symptom combinations, six were very rare, i.e., occurring in less than 0.5 % of the individuals with at least one symptom during their lifetime. This is different from Analysis 1, in which no combinations were found less frequently than 94 times (i.e., in 2.0 % of the 4,682 individuals with at least 1 symptom).

**Fig. 5:**
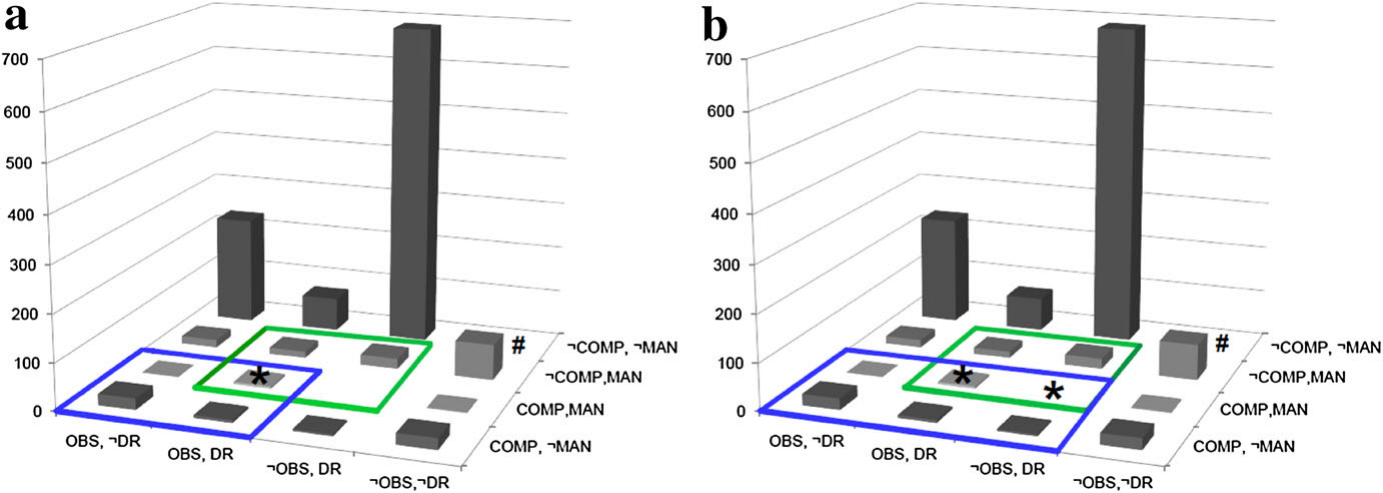
Histogram with responses on “Have you ever suffered from…?” Answers: *OBS* obsessions, *COMP* compulsions, *MAN* manic mood for at least 2 days; *DR* drug use. (* marks comorbidity with rates of 6.9 % in **a** to 6.5 % in **b**) (The column representing the *n* = 5,889 individuals without symptoms has been omitted to improve the visibility of the individuals suffering from one or more symptoms)

As in Analysis 1, two simple monothetic disorders (*D*_*1*_ and *D*_*2*_) are drawn in Fig. 5a. *D*_*1*_ is defined as the combination of obsessions and compulsions (*D*_*1*_: *OBS* ∩ *COMP*); *D*_*2*_ consists of the set manic mood and drugs use (*D*_*2*_: *MAN* ∩ *DR*). In total, 36 patients satisfied *D*_*1*_ and 41 patients had *D*_*2*_. Of those patients, 5 patients satisfied *D*_*1*_ and *D*_*2*_, and thus, suffered from comorbidity. In Fig. 5b, *D*_*1*_ is redefined as the combination of compulsions and obsessions and/or drugs (*D*_*1*_′: *COMP* ∩ (*OBS* ∪ *DR*)). This leads to an extra symptom combination being potentially comorbid, viz., the combination of *COMP*, *MAN*, *DR*. Yet, as this symptom combination does not occur in the sample, the number of comorbidity remains equally low (*n* = 5). Thus, in this case, comorbidity did not increase with a change of diagnosis; the proportion of patients suffering from comorbidity even decreased.

### Conclusions of the analyses

With simple hypothetical diagnoses, we have illustrated that rates of comorbidity depend on the interplay between disease definitions and symptom distributions in populations. Changes in disease definitions sometimes led to changes in comorbidity rates, sometimes not, depending on the prevalence of specific symptom patterns in the population. Neither of those elements in isolation is sufficient to explain rates of comorbidity. Therefore, rates of comorbidity cannot be labeled as resulting either from classification choices, and hence artificial, or from real relations among the diseases, and hence a fact. On the one hand, they are not just an artifact since the rates do depend on a real symptom distribution in a population. On the other hand, comorbidity cannot be said to be a fact independent of human choices, since the rates also depend on choices in disease classification and how the classification captures part of the population.

Of course, our examples feature strongly simplified versions of actual psychiatric disorders, which have been defined in far more intricate and reasoned ways [10, 30]. Moreover, in actual disorders, the symptoms do not behave like the neutral and atomic units that appear in the illustrations. First, the symptoms may have been chosen with the aim of manifesting high correlations. If one is in search of a particular psychiatric disorder, one may be tempted to choose to define symptoms that are often observed together (as a clinical syndrome) and, therefore, are highly correlated. Similarly, and sometimes rather confusingly, symptoms are often definitionally related. For example, muscle tension is only a symptom of GAD if the subject already suffers from anxiety and if the latter symptom is not present, the muscle tension is not included as a symptom. Finally, different questionnaires will target subtly different sets of symptoms, owing to slight variations in how questions are grouped and formulated [31]. For all these reasons, it is not clear that the distribution of symptoms in the population is a crude empirical fact that does not rely on theoretical choices.

We nevertheless believe that the insights from these simple examples apply generally. They hold also for more complex and reasoned definitions of psychiatric disorders and for both latent variable and network models. Moreover, they can be maintained against the background of data sets that are themselves infused with theoretical choices regarding the selection, definition, and operationalization of symptoms. As long as those data rest on empirical input, the resulting measurements in psychiatry will depend simultaneously on the theoretically motivated choices and on the constraints that that empirical input places.

These analyses are a first and rather modest start in explaining the phenomenon of comorbidity. Moreover, it seems to suggest a position in between two extreme views on comorbidity, which we associated with constructivism and realism. However, “in between” is a rather vague indication, and it leaves the problems of circularity and arbitrariness unanswered. In what follows, we go into these problems, and elaborate the “in between” position more precisely.

## Conventionalism about disorders

Above, we argued that comorbidity in psychiatric diagnoses cannot be traced back exclusively to the specifics of the categorization system, nor to the reality of the disorders. Psychiatric comorbidity is a co-production of classification choices and empirical constraints: we determine the set of relevant symptoms and the clustering criteria, but relative to that, the empirical facts, the rates of comorbidity in particular, are manifest. In what follows, we clarify that this particular view on comorbidity exemplifies a more general idea on the relation between scientific theory and empirical fact, which has a long history in the philosophy of science: conventionalism [14, 15]. We make the idea of conventionalism more precise by first discussing the problems of the circularity and arbitrariness of disease classification, as mentioned earlier. Conventionalism puts these problems in a different light.

### Definitional circularity and arbitrariness

Broadly speaking, psychiatric disease classifications perform a double function. On the one hand, the DSM can be viewed as a theory about the psychiatric realm, meaning, the classification serves to represent the subject matter of psychiatry. On the other hand, it also serves as a tool for diagnosing psychiatric disorders, that is, as a device used for measurement and not as a structure used for representation. In what follows, we make precise how the double function becomes problematic, and indicate how a conventionalist position resolves the issues.

Notice that the general opposition between a realist and constructivist perspective on disease classifications works out differently for the two functions just outlined. Consider the DSM as a theory about the psychiatric realm. The opposition concerning the status of the DSM then runs parallel to the opposition between realist and constructivist leanings in ontology: a realist would say that terms from the DSM are then taken to refer to an independent reality of mental disorders, and the DSM might describe these disorders more or less truthfully; a constructivist would say that terms from the DSM are projected onto the phenomena of psychiatry, and so provide structure to these phenomena [32].

Now, consider the DSM as a tool for diagnosing psychiatric disorders. The same opposition between realist and constructivist ideas then obtains a more epistemological reading, and runs parallel to a well-known opposition from the philosophy of experiment (cf. [16]): either the DSM facilitates a *representation* of psychiatric phenomena, i.e., providing passive epistemic access to the phenomena, or it is better seen as involved in the *production* of psychiatric phenomena, i.e., actively creating the phenomena. Clearly, these oppositions concern the same underlying tension, which arguably permeates the whole of science: should one trace scientific knowledge back to an independent reality, or is it rather the result of our own epistemic doing?^10^

An argument that is used to resolve this tension in favor of the realist camp in some domains is sometimes called “bootstrap confirmation” (e.g., [34]). A theory is more likely to describe an independent structure if it is supported by results from a measurement device that relies on an entirely different theory. In turn, a measurement device is more likely to provide a neutral representation of a phenomenon if it also works to confirm theories that pertain to entirely different phenomena. Take for example an MRI scanner, which is developed by means of physics but can be used to support theories in neuroscience. It is unlikely that systematic errors in how the scanner works are such that they become misleading for the neuroscientists. And even if one runs this risk, one can check for systematic errors or calibrate the scanner by relying on physics.^11^ The problem for the DSM is that measurement device and theory coincide exactly.

This is the exact point where the definitional circularity referred to earlier becomes a seemingly vicious one, and where the so-called experimenter’s regress becomes a pressing issue (cf. [35]). In that regress, the experimenter defines what she observes by reference to the correct method of observing it, but she also defines the correct method by reference to what she is supposed to observe. For example, an experimenter might say temperature is that which is measured by a thermometer, and then add that a thermometer is any device that measures temperature. Something similar seems to occur for mental disorders: we say that MDD can be measured by checking for a set of nine symptoms (thus, the nine symptoms serve as a measurement tool), but then our use of those symptoms for identifying MDD is motivated by reference to MDD as a pre-given mental disorder (thus, the nine symptoms serve as a theoretical structure).

The usual resolution to this is to find different methods of observing the same phenomenon, i.e., *triangulation*, or else to find different phenomena to which to apply the same method of observation, i.e., *calibration*. Both lead to an independent check of the measurement procedure at stake. However, in our research of mental illness, we cannot calibrate the use of a tool for one theory by relating it to another, and neither can we triangulate the theory by finding two different tools that provide independent support. Instead, we are left with a theoretical structure that doubles as its sole measurement device. It seems inevitable that this device provides measurement outcomes that fit the theoretical structure. So, we are led to the conclusion that the whole schema, consisting of both theory and tool, is of our own doing, i.e., an arbitrary construction that is imposed on reality rather than a structure uncovered in it.

### Resolution: coordinative principles

Conventionalism offers an escape from this circularity, and thereby, presents an alternative to the conclusion that disease classifications are merely constructions imposed on the phenomena. Admittedly, a number of conventions regarding disorders and their structure cannot be avoided. Access to the structure of mental disorders cannot be gained by means other than diagnostic tools or measurement devices that provide some structure themselves. For example, a set of nine symptoms is used as an indication of the disorder “depression.” But as psychiatrists, we have no other way of determining whether or not someone suffers from depression than by finding out if they have at least five out of those nine symptoms. Effectively, we stipulate that those nine symptoms effectively are constitutive of depression.

Our point is that such stipulations, or, more appropriately, coordinative principles, improve our grip on the subject matter of psychiatry. Coordinative principles are bridge principles that coordinate concepts (e.g., depression) to empirical reality (members of the population with a certain symptom profile). In short, they fix the empirical content of concepts. The principles are necessary conditions to start organizing the empirical facts by means of the concepts, and thus serve as a basis for gaining knowledge about psychiatric disorders [15]. The definition of the disorders occasions the expression of associations that would otherwise be very hard, if not impossible, to pin down. Once certain concepts are stipulated, like MDD and GAD, specific patterns will become apparent in the measurement results. And these patterns do convey something genuine and informative about the world of psychiatric phenomena. In terms of our example, it so happens that MDD shows strong correlations with GAD. While the association of MDD and GAD may be partly due to stipulations, there is evidently some empirical fact to which this association can be traced back. After all, the opposite could also have been found: that MDD and GAD are negatively correlated, or not correlated at all. Thus, measurement results that are couched in terms of the DSM reveal something genuine about psychiatric disorders.

With this in mind, we return to the—possibly vicious—circularity in the study of mental disorders. Given the current state of psychiatric science, we do not have an independent way of verifying that a subject indeed suffers from MDD, so as to anchor or substantiate the conventional choices that define depression. But we need not do so. We can employ the convention that particular symptoms constitute depression as a basis for gaining empirical knowledge. The fact that depression is constituted by nine symptoms is not itself a substantive claim about the world of psychiatric phenomena, which it could be if we had some way of resolving the circularity, e.g., some other epistemic access to depression than through those symptoms. The point is that this convention, or coordinative principle, occasions substantive claims about mental disorders, some of which could otherwise not be made. For example, one can claim that antipsychotics in combination with antidepressants are more effective than monotherapy with either antipsychotics or antidepressants in treating psychotic depression [36], because one has laid down a useful convention about what constitutes psychotic depression.

These substantive claims provide a way out of the vicious circularity and arbitrariness of mental disorders. Obviously, some definitions of mental disorders will be more successful in occasioning those substantive claims than others. Because of this, the coordinative principles are more than eliminable shorthands for more complicated relations that obtain among the symptoms. Some principles chime better than others with the empirical patterns on which they rest. Similarly, some principles track the causal structure of psychiatric phenomena better than others. In other words, a conventionalist interpretation of psychiatric disorders does not amount to an “anything goes” attitude: not any random collection of symptoms constitutes a useful disease classification. Because of the variation in more and less successful substantive claims that may follow coordinative principles, we escape the conclusion that the whole edifice of psychiatric diagnosis is self-congratulatory and subjective. Though the coordinative principles cannot be true or false—they are mere conventions—something can be objectively right about them.^12^

### Conventionalism

We now explain the foregoing perspective on disorders by falling back on long-standing ideas about *conventionalism* and coordinative principles in the philosophy of science. Examples are abundant: the nature of temperature vis-à-vis the status of the thermometer, the nature of color as a physical phenomenon and as expressed by a color space, and the nature of physical space in relation to the status of our mathematical models of it [16, 35]. It is illuminating to relate the foregoing to this broader debate.

We briefly focus on conventionalism about space and time in physics. Following the received view on conventionalism [15], there is no objective fact as to what constitutes a straight line in physical space. A straight line is a mathematical notion, whereas physical space is presumably “out there,” as a coordinate system for objects or perhaps even as a substance. It is not given in advance how the mathematical notions are supposed to be applied to physical space. This is rather something that needs to be stipulated, or laid down in conventions or coordinative principles. However, once the trajectory followed by a freely falling test particle is associated with the mathematical concept of a geodetic curve, various other claims about geodetic curves become substantive, and in fact, highly informative. For instance, owing to certain conventions, one can claim that light follows such geodetic curves and so is deflected in a gravitational field. Notably, this is achieved without calibrating the trajectory of the freely falling body or triangulating the geometry in which the geodesics are described. Neither of these two even makes sense because geodesic and trajectory are associated by convention. The conventions themselves do not amount to claims that may be true or false. Nevertheless, the convention occasions substantive claims about the geometry of physical space, which can be true or false. Moreover, some systems of conventions are clearly more economical or successful than others.

Our suggestion is that psychiatric disease classifications are conventions in much the same way. Notice the specific meaning that is attached to the notion of convention here. They help to coordinate a theoretical structure with an empirical one, and they vary in how successful they are at that. So the term “convention” should certainly not give the impression that “anything goes.” Moreover, in the case of psychiatric classifications as well as of physical geometry, care is needed in interpreting claims that are here called substantive and which, in the vocabulary employed earlier in this article, express something real, genuine, or robust about the subject matter. These terms are not intended to signal that all the notions employed have their referent in some realist world picture. No such position in the spectrum between scientific realism and empiricism is implied by the substantiveness of the claims. What is meant is that substantive claims eventually find their basis in something other than the conventions, be it some empirical patterns or a principle or mechanism underlying those patterns. So, in the case of our example about claims concerning the comorbidity of MDD and GAD as revealing something genuine about these psychiatric disorders, what is meant is that their comorbidity cannot be traced back in its entirety to conventions adopted to delineate these disorders. Their comorbidity points to something genuine, be it strictly on the level of empirical fact or on the level of causal relations.

Our proposal to view psychiatric disease classifications as conventions is by no means intended as a full-fledged theory of what disease classifications are, or as a rival to extant accounts of the ontological and epistemological status of mental disorders (see e.g., [33]). We do not opt for any specific realist, anti-realist, or constructivist viewpoint by proposing conventionalism. Moreover, in this article, we say little about the way in which conventions are chosen and evaluated by users of a theory. We believe that pragmatic considerations, which direct our choices for scientific theories and models [37], could be central to the choice of conventions too, but we do not argue for this in this article. Here, we merely propose and illustrate a particular view on comorbidity, and its reflection on psychiatric disease classification more generally. An account of how this might transform the debate over the status of disease classifications is beyond the scope of this article.

That said, our proposal counters extreme positions in the spectrum of realism and constructivism. A theorist with strong constructivist sympathies might frown upon the suggestion that there is anything “genuine” without the support of construction work. And working psychiatrists in turn might frown upon philosophers who debate the reality of disorders that they are confronted with on a daily basis, and which exert such real causal power over people. We invite both sides to approach the issues in a relaxed mood. Independently of the ontological status eventually given to mental disorders, we argue that their structure can only be captured after laying down conventions. Those conventions are located somewhere outside the force field between constructivism and realism.^13^

## Conclusion

So, how should psychiatric comorbidity be interpreted, and what does it illustrate about psychiatric disorders? While some emphasize the constructivist character of this phenomenon, pointing to classification choices in the DSM, others stress the reality of comorbidity, pointing to underlying causal mechanisms. We showed by empirical and conceptual arguments that both positions are insufficient to account for comorbidity. We then argued for a conventionalist approach: rates of comorbidity depend on the interplay between classification choices and empirical reality, and classifications in psychiatry are best seen as coordinative principles. Importantly, this does not take away the fact that those classifications may occasion an objective, informative, and non-arbitrary description of psychiatric reality.

As we argued, the debate on comorbidity echoes realist and constructivist intuitions about psychiatric disorders in general. But broadly speaking, both positions ignore important aspects of psychiatric disorders. On the one hand, a realist view commits us to the actual existence of the entities and structure of psychiatric disorders, and thus, neglects the relativity of measurement results based on DSM classifications. The realist idea that current disorders refer to the real structure in the world might thus lead to hasty reification, and enhance the search for causal mechanisms and treatments for extant disorders, without taking into consideration the adequacy of the classifications themselves. On the other hand, a constructivist position entails that the DSM categories “make” psychiatric diseases, which leads to sharp attacks on the idea that psychiatric disorders are real. Thus, constructivists pass over the fact that robust syndromes have occurred in the psychiatric domain long before the introduction of the DSM, as for instance, depression [40].

We submit that a conventionalist position fits better with psychiatric reality, as it acknowledges the relativity of DSM classifications while at the same time recognizing the objectivity and wealth of knowledge based on those classifications. It can easily deal with the fact that different versions of the DSM lead to different measurement results without discarding the mind-independent character of those measurements. Furthermore, conventionalism is suited to handle the problems of the circularity and arbitrariness of symptom sets. The fact that coordinative definitions precede the acquisition of empirical knowledge does not lift all demands from those definitions. Coordinative definitions themselves are subject to all kinds of constraints, often of a pragmatic nature, e.g., coherence and usefulness. By way of comparison, the development of the thermometer shows that there were once clear reasons to alter the definition and measurement of temperature, despite the close connection between theory and measurement device [35].

In a similar vein, there are criteria that escape the circularity and arbitrariness of current psychiatric diagnoses. Diagnoses could, for instance, be assessed in terms of their success in coinciding with a causal background and increasing understanding, in predicting course and outcome, and in guiding treatment decisions. Another direction worthwhile in the evaluation of psychiatric diagnoses is to take a closer look at symptom distributions in a population, and how they are caught by disease models, for instance, by elaborating the simple analyses we performed above on the NEMESIS study. Mapping the symptom distributions in a population might provide insight into the extent to which DSM-models catch discrete disorders in terms of symptoms, or whether they are not separated by “zones of rarity.” We take this point as a valuable contribution to the philosophical and methodological debate over psychiatric disorders. Our position embraces the conventional aspect of the DSM and utilizes this to improve its applications, without robbing the DSM of its mind-independent content.

We realize that the evaluation of psychiatric disease models is incredibly complex. First, many criteria are important in evaluating their usefulness, of which the description of a discrete set of symptoms is only one. Other criteria such as the possibility to interfere, the ability to track causal mechanisms, or the reliability in diagnosing patients are important concerns, and do not necessarily improve with diseases as a discrete set of symptoms. Second, what is the case for one DSM diagnosis does not necessarily apply to all DSM diagnoses. On the contrary, ADHD, for example, may function very differently from bipolar disorder on many of the criteria mentioned above. A third factor complicating the evaluation of psychiatric disease models is the major impact of the DSM on society, politics, and the pharmaceutical industry. All these factors together make the evaluation of psychiatric disease models a challenging enterprise.

So, what are the benefits of all this? There is and has been a lot of debate on the interpretation of comorbidity and on the conceptualization of psychiatric diseases more generally. Central in this debate has been the question: what kind of things are psychiatric disorders? With our conceptual clarification, we have aimed to propose a perspective that gives disease definitions a different status, and so frees up research into alternative classifications. We hope future research will aim at investigating the strengths and weaknesses of each specific disease model, and thus move psychiatry forward.

## Footnotes

1 In what follows, we will use the terms “mental disorder,” “psychiatric disease,” “mental illness,” and permutations thereof interchangeably, as is custom in the literature.

2 Similar conclusions hold for mental disorders described in the International Classification of Disease [17].

3 It is not inherent to a latent variable approach that the latent variables function as entities and obtain a causal role, but often researchers do interpret the latent variables in this way [20].

4 Both positions in the debate agree that causal disease models are preferable to non-causal disease models [23]. The disagreement is in the signal that comorbidity is supposed to give: either it suggests that our current disorders are fuzzy symptom sets without any relation to causal structures and need to be replaced by completely new definitions, or it suggests that there are causal links between our current disorders, and therefore the current diagnoses should be integrated in a more general causal structure.

5 The problem is similar to the experimenter’s regress, which has been found more broadly in science (cf. [26]). As will be argued further down, we think that the regress is particularly pressing here.

6 This is a rather coarse description of the positions one might take towards psychiatric disease classification. In section “Comorbidity as dependent on causality” we will be more specific on the philosophical views against which our own views are offset.

7 For reasons of simplicity, we assume independence of these two elements in the sense that a change in the disease definition will not lead to a change in the symptom distribution in a population, although we acknowledge that such a change might influence a distribution of symptoms [27]. The independence is not required for our argument.

8 The diagrams are known as Karnaugh maps [28].

9 The combination of drug use and obsessions was more prevalent than the combination of obsessions and compulsions, which might be due to the relatively high lifetime prevalence of drug use as opposed to the low lifetime prevalence of compulsions.

10 We realize that the philosophy of psychiatry provides a more nuanced picture of the tension between realism and constructivism (see, e.g., [33]). But for the purposes here—clarifying a particular intermediate perspective on classification—it is not necessary to spell out this tension in detail.

11 Following a remark by Duhem (1906), psychiatry is not the only scientific discipline unable to test theories by means of bootstrap confirmation or triangulation: “the experimental testing of a theory does not have the same logical simplicity in physics as in physiology.” In physiology, theories are tested by means of laboratory instruments that are based on theories of physics, but in physics, “it is impossible to leave outside the laboratory door the theory we wish to test” (cited in Chang [35, p. 221]).

12 We might say that a classification system is thereby triangulated, or calibrated: once a classification choice allows us to relate a diverse set of psychiatric and somatic phenomena, we might say that this choice has itself been confirmed. However, we think that it is not appropriate to view classification choices as substantial claims that can be confirmed, supported, or falsified by empirical fact. Conventions have quality criteria, but they should not be viewed as truth conditions.

13 A very similar middle position has been developed by Kuipers under the name of “constructive realism” [38]. Our ideas have been inspired by Kuipers’s position that reality is best understood as a co-production of subject and object, which itself may be likened to a relativized or dynamic Kantian view [39].
